# Patterns of Suya (roasted beef) consumption, awareness of trace metal contamination, and food safety practices among residents of Yenagoa Metropolis, Nigeria

**DOI:** 10.3389/fpubh.2026.1779695

**Published:** 2026-03-20

**Authors:** Sylvester Chibueze Izah

**Affiliations:** Department of Community Medicine, Faculty of Clinical Sciences, Bayelsa Medical University, Yenagoa, Bayelsa, Nigeria

**Keywords:** food safety awareness, knowledge and perception, Nigeria, public health, street-vended meat, Suya consumption, trace metal contamination

## Abstract

**Background:**

Suya, a widely consumed street-vended meat in Nigeria, may expose consumers to trace metal contamination due to informal preparation and vending conditions. Given its frequent patronage, contamination risk is shaped not only by exposure but also by how often people consume Suya, the factors influencing vendor choice, and their understanding of contamination and protective behaviors. In this study, “knowledge” refers to consumers' understanding of trace metal sources and health effects, while “awareness/safety practices” reflect risk perception and behavioral responses to minimize exposure. Therefore, this study assessed socio-demographic characteristics, consumption behaviors, knowledge of trace metal contamination, and safety practices among Suya consumers in Yenagoa metropolis, Nigeria.

**Methods:**

A cross-sectional survey was conducted between March and May 2024 among 396 regular Suya consumers selected through time-location-based simple random sampling across markets, schools, residential areas, and evening vending spots to reflect varying consumption periods. Eligible participants were residents aged ≥16 years who had consumed Suya within the previous 3 months to ensure stable and recent consumption patterns. Data were collected using a structured questionnaire. The response rate was 92.7%. Data were analyzed using SPSS version 21. Frequencies, percentages, and composite scores were computed. Associations were tested using Pearson's Chi-square. Internal consistency was evaluated with Cronbach's alpha (overall α = 0.75) and Spearman's rank correlation examined relationships among section scores.

**Results:**

Most respondents were female (71.5%), aged 16–25 years (66.2%), single (79%), and had tertiary education (71.7%). Suya consumption was frequent, with 58.3% buying from roadside vendors; age was significantly associated with vendor choice (*p* = 0.003). Occupation influenced availability near workplace/school (*p* = 0.036). Education was significantly associated with higher-level contamination knowledge and regulatory support. Positive correlations were observed between consumption patterns and influencing factors (ρ = 0.546, *p* < 0.01) and between knowledge and safety practices (ρ = 0.132, *p* < 0.01).

**Conclusion:**

Suya consumption is shaped by accessibility and convenience, while education enhances contamination knowledge and protective behaviors. Targeted public health education and strengthened vendor monitoring are recommended to reduce potential trace metal exposure.

## Introduction

Street-vended foods constitute a critical component of urban diets in Nigeria, providing millions of residents with affordable, convenient, and culturally significant meals. In Bayelsa State, commonly consumed street foods include groundnuts (1); snacks such as puff-puff and meat pies (2, 3); fresh fruits, including watermelon, orange, pineapple, apple, and cucumber (4, 5); and fruit-based beverages such as zobo, kunu, soya, and tiger nut drinks (6–8). Among these options, Suya, a spiced grilled meat, stands out for its widespread popularity, driven by its flavor, ease of access, and minimal preparation requirements. Its convenience makes it particularly appealing to individuals with busy schedules, students, and urban dwellers seeking ready-to-eat protein sources. However, despite its popularity, concerns about food safety have intensified, particularly regarding chemical contaminants such as trace metals (9, 10). Metals, including lead, cadmium, chromium, and nickel, can accumulate in animal tissues, such as those of cows and fish, through environmental exposure or unhygienic handling practices (11–18). Long-term exposure to these metals can result in serious health effects, including kidney and liver damage, neurological disorders, and cancer (19). These risks highlight the public health significance of monitoring and regulating street-vended foods such as Suya.

Multiple socio-demographic and environmental factors shape consumer behavior related to Suya consumption. Younger adults, particularly students, constitute a large share of street food consumers, often prioritizing taste, convenience, and affordability over hygiene and safety. Educational attainment has been shown to influence both the depth of knowledge and risk perception, with more educated individuals demonstrating greater awareness of contamination risks and more substantial support for regulatory oversight. However, basic food safety awareness is widely shared across educational levels. Other socio-demographic factors, including occupation, marital status, and cultural practices, also shape consumption patterns. This is particularly evident in urban settings, where Suya vendors cluster around schools, workplaces, and residential areas, increasing access and convenience for consumers. Such environmental and social factors reinforce consumption behaviors, sometimes at the expense of safety considerations.

Despite widespread Suya consumption, limited research in Nigeria has examined the interrelationships among consumption patterns, influencing factors, knowledge of trace-metal contamination, and safety practices among consumers. Most studies have focused primarily on microbial and chemical contamination, with an emphasis on trace metals (10, 20). On a broader scale, food safety research often examines either vendor practices or general consumer awareness (21–25), without adequately linking these outcomes to socio-demographic characteristics or behavioral drivers. Understanding these relationships is therefore critical for designing targeted public health interventions, regulatory monitoring, and consumer education programs, all of which can help reduce exposure to contaminated foods and promote safer street food consumption practices.

This study, therefore, aimed to assess the socio-demographic profiles of Suya consumers, document their consumption patterns, identify factors influencing consumption, evaluate knowledge of trace metal contamination, and examine awareness and safety practices among residents of Yenagoa metropolis, Bayelsa State, Nigeria. By linking socio-demographic factors to consumption behaviors and safety practices, the study provides evidence to inform public health interventions, regulatory monitoring, and consumer education programs, ultimately contributing to safer street-vended foods in urban Nigerian settings. The findings also shed light on the behavioral drivers of Suya consumption and highlight gaps between knowledge and practice, underscoring the need for integrated approaches to minimize the health risks associated with trace metal exposure.

## Methodology

### Study area

The study was conducted in Yenagoa metropolis, Bayelsa State, Nigeria, the state's administrative and economic center. The city has a diverse population composed of students, traders, civil servants, and informal workers, making it an ideal location to study patterns of Suya consumption, factors influencing consumption, and awareness of food safety. Suya is widely consumed in Yenagoa, available at roadside vendors, markets, and social gatherings, providing a practical context for evaluating consumption behaviors and associated health risks.

### Study design

A cross-sectional descriptive survey design was employed to obtain information about the consumption patterns, influencing factors, knowledge of trace metal contamination, and awareness/safety practices among residents. The study was conducted between September and December 2025. The cross-sectional design allowed for the collection of data at a single point in time to assess the prevalence of Suya consumption behaviors and associated factors without manipulating study variables.

### Sample size determination

Cochran's formula, previously described by Ogbeibu (26), was adopted for the study, since the metropolis's population exceeds 10,000. Furthermore, information on previous prevalence for this topic is scarce in the literature; hence, a conservative prevalence (*p* = 0.5) was used for the study. The confidence level of 95% (Z = 1.96) and the margin of error (d = 0.05) were adopted.


$$\begin{array}{lll} n & = & \frac{Z^{2}\;x\;P\;x\;\left(1-p\right)}{d^{2}}\\ n & = & \frac{1.96^{2}\;x\;0.5\;x\;\left(1-0.5\right)}{0.05^{2}}=\frac{3.8416\;x\;0.25}{0.0025}=\frac{0.9604}{0.0025}=384.16 \end{array}$$

Adjusting for the non-response rate of 10%


$$\begin{array}{l} nadj=\frac{n}{1-Non-reponse\;rate}\text{=}\frac{384.16}{1-0.10}=\frac{384.16}{0.90}\\ \;=426.82=427. \end{array}$$

Of these, only 396 questionnaires were retrieved, accounting for 92.74% and exceeding the exact sample size.

### Sampling technique

A simple random sampling technique was used to select participants. To account for variation in Suya consumption patterns across time and location, a time-location sampling framework was first developed. High-consumption points (markets, schools, residential clusters, roadside evening vending areas) were identified through preliminary field mapping conducted 1 month prior to data collection. Sampling was then conducted during peak consumption periods (afternoon and evening hours) as well as off-peak periods, including both weekdays and weekends. At each location, individuals who met the inclusion criteria were enumerated or approached, and respondents were selected using a random selection procedure such as balloting without replacement or a random number table until the required sample size was achieved. This procedure ensured that sampling reflected real-world consumption patterns and minimized temporal and spatial selection bias.

### Inclusion and exclusion criteria

#### Inclusion criteria

Residents aged ≥16 years who had consumed Suya within the preceding 3 months and provided informed consent were included. The 3-month criterion was adopted to ensure participants had recent and stable consumption experience, consistent with behavioral nutrition research indicating that recall reliability and habitual dietary assessment are strongest within a 1–3 month reference period.

#### Exclusion criteria

Individuals with dietary restrictions or medical conditions **(**food allergies to Suya ingredients, vegetarian or vegan diets, religious or cultural dietary restrictions, gastrointestinal disorders aggravated by spicy foods, chronic medical conditions requiring dietary control) that prevent Suya consumption were excluded from participation. In addition, potential respondents who declined or were unable to provide informed consent were excluded from the study.

### Questionnaire development

Data were collected using a structured, self-administered questionnaire specifically designed to capture Suya consumption patterns, influencing factors, knowledge of trace-metal contamination, and awareness/safety practices. The questionnaire was developed based on a review of relevant literature on food consumption behaviors, food safety, and trace metal contamination, as well as guidance from experts in nutrition, public health, and environmental health. The instrument was divided into two parts Part 1 (sociodemographic characteristics of the respondents) and Part 2. The Part 2 have four sections, including Section A (Suya Consumption Patterns), Section B (Factors Influencing Consumption), Section C (Knowledge of Trace Metal Contamination), and Section D (Awareness, Perception, and Safety Practices). All items were measured on a five-point Likert scale ranging from 1 = Strongly Disagree to 5 = Strongly Agree, except for the respondents‘ socioeconomic characteristics (Supplementary material). Typically, “Knowledge” items assessed factual understanding of contamination sources and health effects, whereas “awareness/safety practice” items measured perceived risk, regulatory expectations, and protective behavioral intentions. Furthermore, “awareness” refers to respondents' level of concern and recognition of potential contamination risks and regulatory responsibilities; “perception” reflects their attitudes toward contamination severity and trust in food safety oversight; while “safety practices” represent self-reported behavioral intentions or actions taken to reduce potential exposure (e.g., reducing intake, reheating, preferring certified vendors). The questionnaire in Google form was administered in English, and where necessary, clarifications were provided in the local language to enhance comprehension. Completion time ranged between 10 and 15 min.

### Validation and reliability

The questionnaire was pre-tested on 20 respondents in Amassoma, Bayelsa State, a community with socio-demographic characteristics similar to Yenagoa but outside the study area. The pre-test assessed item clarity and relevance, and ambiguous questions were revised based on participant feedback and researcher observations. Pre-test data were analyzed for internal consistency using Cronbach's alpha; values >0.75 overall (sub-sections 0.70–0.79). The value has acceptable internal consistency, confirming that the items reliably measure their intended constructs (27).

Pre-testing was conducted among 20 respondents in Amassoma. Ambiguous items were revised. Pre-test Cronbach's alpha was >0.75 (subsections 0.70–0.79).

### Composite score calculation

The composite score was calculated through a systematic procedure. First, responses to each questionnaire item were measured on a five-point Likert scale and numerically coded in ordinal order, with Strongly Disagree coded as 1, Disagree as 2, Neutral as 3, Agree as 4, and Strongly Agree as 5. Secondly, item-wise mean scores were computed for each questionnaire item within each section. This was done by summing the scores of all respondents for a given item and dividing the total by the number of respondents.

Furthermore, the composite mean score was interpreted using predefined decision criteria. Mean scores ranging from 1.00 to 2.33 were classified as Disagree, scores between 2.34 and 3.67 were classified as Neutral, and scores from 3.68 to 5.00 were classified as Agree, as adopted from Ramli et al. (28).

### Statistical analysis

Data were analyzed using SPSS version 21 (IBM Corp., Armonk, NY, USA). Descriptive statistics, including frequencies and percentages, summarized socio-demographic characteristics, Suya consumption patterns, influencing factors, knowledge, and safety practices. Pearson's Chi-square tests were used to examine associations between demographic variables and responses to individual items—for example, age for Section A (Suya Consumption Patterns), occupation for Section B (Factors Influencing Consumption), and education level for Sections C (Knowledge of Trace Metal Contamination) and D (Awareness, Perception, and Safety Practices). Spearman's rank correlation was conducted using composite scores to assess relationships among consumption patterns, influencing factors, knowledge, and safety practices. Cronbach's alpha was computed to determine the reliability of the final questionnaire. A p-value <0.05 was considered statistically significant. Prior to inferential analysis, data were screened for completeness and accuracy. The assumptions for Chi-square (expected cell count ≥5) and Spearman's correlation (ordinal/continuous composite scores and monotonic relationship) were verified before analysis. All statistical tests were two-tailed.

## Results and Discussion

### Socio-demographic characteristics of respondents

The study included 396 respondents from Yenagoa metropolis, whose socio-demographic characteristics are summarized in Table 1. Gender distribution showed that the majority were female (71.5%) compared to 28.5% males. This suggests that women are more actively engaged in Suya consumption and related food practices. This also highlights the prominent role of women in household food choices and consumption behavior.

**Table 1:** Socioeconomic characteristics of the respondents and awareness of Suya contaminants.

**Questions**	**Categories**	**Frequency (*n*)**	**Percent (%)**
Gender	Male	113	28.5
	Female	283	71.5
	Total	396	100.0
Age	16–25 Undergraduate	262	66.2
	≥ 25 adults	105	26.5
	16–49 Pregnant/lactating women	29	7.3
	Total	396	100.0
Marital status	Single	313	79.0
	Married	81	20.5
	Widowed	2	0.5
	Total	396	100.0
Educational Level	No primary education	0	0
	Primary	0	0
	Secondary	13	3.3
	Tertiary	284	71.7
	Postgraduate	99	25.0
	Total	396	100.0
Occupation	Student	280	70.7
	Trader	5	1.3
	Civil Servant	76	19.2
	Artisan	2	0.5
	Unemployed	10	2.5
	Others	23	5.8
	Total	396	100.0
Religion	Christianity	392	99.0
	Islam	4	1.0
	Traditional	0	0
	Total	396	100.0

The age profile revealed that most respondents were 16–25-year-old undergraduates (66.2%), followed by adults aged ≥25 years (26.5%), with a smaller proportion of pregnant and lactating women aged 16–49 years (7.3%). The predominance of younger participants reflects urban consumer trends in Nigeria, where students and young adults often represent a significant segment of street food consumers (29). Regarding marital status, 79.0% of respondents were single, 20.5% were married, and 0.5% were widowed. The high proportion of single individuals indicates a population likely to make independent food choices, potentially influencing consumption patterns and safety practices.

Educational attainment showed that none of the respondents had primary and no formal education; 3.3% had secondary education; 71.7% had tertiary education; and 25.0% had postgraduate qualifications. The high level of education suggests that most respondents are likely to be aware of health risks and food safety issues. This may positively affect their hygiene and consumption practices.

In terms of occupation, students were the majority (70.7%), followed by civil servants (19.2%), traders (1.3%), artisans (0.5%), unemployed individuals (2.5%), and other occupations (5.8%). This distribution indicates that Suya consumption is widespread among students, who may rely on convenient street food options. Religion was predominantly Christian (99.0%), with a small proportion practicing Islam (1.0%). Religious affiliation can influence dietary habits and perceptions toward food safety, potentially shaping how consumers engage with street-vended foods.

## Association between age category and Suya consumption behaviors

Table 2 presents the association between age category (16–25 undergraduates, ≥25 adults, and 16–49 pregnant/lactating women) and Suya consumption behaviors among 396 respondents in Yenagoa metropolis. Weekly consumption of Suya did not differ significantly across age groups (χ^2^ = 12.157, *p* = 0.144). Overall, 66.9% of respondents either strongly disagreed and disagreed that they consume Suya at least once a week, indicating that Suya consumption is generally occasional rather than habitual. This pattern was relatively consistent across the three age categories.

**Table 2:** Association Between Age Category (16–25 Undergraduates, ≥25 Adults, and 16–49 Pregnant/Lactating Women) and Suya Consumption Behaviors in Yenagoa Metropolis, Nigeria.

**Question**	**Age group**	**SD *n* (%)**	**D *n* (%)**	**N *n* (%)**	**A *n* (%)**	**SA *n* (%)**	**Total**	**χ^2^**	***p*-value**
Consume Suya at least once a week	16–25 Undergraduate	76 (29.0)	89 (34.0)	49 (18.7)	39 (14.9)	9 (3.4)	262	12.157	0.144
	≥25 Adult	45 (42.9)	32 (30.5)	15 (14.3)	8 (7.6)	5 (4.8)	105		
	16–49 Pregnant/lactating	13 (44.8)	10 (34.5)	4 (13.8)	2 (6.9)	0 (0.0)	29		
	Total	134 (33.8)	131 (33.1)	68 (17.2)	49 (12.4)	14 (3.5)	396		
Buy from roadside vendors	16–25 Undergraduate	14 (5.3)	8 (3.1)	25 (9.5)	166 (63.4)	49 (18.7)	262	23.307	0.003
	≥25 Adult	12 (11.4)	4 (3.8)	9 (8.6)	53 (50.5)	27 (25.7)	105		
	16–49 Pregnant/lactating	8 (27.6)	0 (0.0)	2 (6.9)	12 (41.4)	7 (24.1)	29		
	Total	34 (8.6)	12 (3.0)	36 (9.1)	231 (58.3)	83 (21.0)	396		
Eat Suya at night (6–9 PM)	16–25 Undergraduate	18 (6.9)	10 (3.8)	21 (8.0)	156 (59.5)	57 (21.8)	262	12.888	0.116
	≥25 Adult	12 (11.4)	5 (4.8)	8 (7.6)	48 (45.7)	32 (30.5)	105		
	16–49 Pregnant/lactating	6 (20.7)	1 (3.4)	1 (3.4)	13 (44.8)	8 (27.6)	29		
	Total	36 (9.1)	16 (4.0)	30 (7.6)	217 (54.8)	97 (24.5)	396		
Prefer Suya over other meat	16–25 Undergraduate	76 (29.0)	94 (35.9)	62 (23.7)	25 (9.5)	5 (1.9)	262	6.543	0.587
	≥25 Adult	38 (36.2)	36 (34.3)	17 (16.2)	11 (10.5)	3 (2.9)	105		
	16–49 Pregnant/lactating	10 (34.5)	10 (34.5)	5 (17.2)	2 (6.9)	2 (6.9)	29		
	Total	124 (31.3)	140 (35.4)	84 (21.2)	38 (9.6)	10 (2.5)	396		
Consume more than other meat snacks	16–25 Undergraduate	98 (37.4)	80 (30.5)	33 (12.6)	42 (16.0)	9 (3.4)	262	5.158	0.741
	≥25 Adult	41 (39.0)	35 (33.3)	7 (6.7)	17 (16.2)	5 (4.8)	105		
	16–49 Pregnant/lactating	11 (37.9)	8 (27.6)	5 (17.2)	3 (10.3)	2 (6.9)	29		
	Total	150 (37.9)	123 (31.1)	45 (11.4)	62 (15.7)	16 (4.0)	396		
Buy based on taste vs. hygiene	16–25 Undergraduate	30 (11.5)	51 (19.5)	66 (25.2)	95 (36.3)	20 (7.6)	262	12.591	0.127
	≥25 Adult	24 (22.9)	22 (21.0)	23 (21.9)	29 (27.6)	7 (6.7)	105		
	16–49 Pregnant/Lactating	8 (27.6)	5 (17.2)	6 (20.7)	7 (24.1)	3 (10.3)	29		
	Total	62 (15.7)	78 (19.7)	95 (24.0)	131 (33.1)	30 (7.6)	396		
Consume more during weekends	16–25 Undergraduate	50 (19.1)	89 (34.0)	78 (29.8)	40 (15.3)	5 (1.9)	262	11.015	0.201
	≥25 Adult	26 (24.8)	28 (26.7)	28 (26.7)	19 (18.1)	4 (3.8)	105		
	16–49 Pregnant/lactating	6 (20.7)	10 (34.5)	8 (27.6)	2 (6.9)	3 (10.3)	29		
	Total	82 (20.7)	127 (32.1)	114 (28.8)	61 (15.4)	12 (3.0)	396		
Patronize same vendor	16–25 Undergraduate	17 (6.5)	45 (17.2)	52 (19.8)	121 (46.2)	27 (10.3)	262	23.668	0.003
	≥25 Adult	18 (17.1)	31 (29.5)	14 (13.3)	33 (31.4)	9 (8.6)	105		
	16–49 Pregnant/lactating	5 (17.2)	8 (27.6)	6 (20.7)	8 (27.6)	2 (6.9)	29		
	Total	40 (10.1)	84 (21.2)	72 (18.2)	162 (40.9)	38 (9.6)	396		
Consume because readily available	16–25 Undergraduate	28 (10.7)	85 (32.4)	67 (25.6)	72 (27.5)	10 (3.8)	262	10.783	0.214
	≥25 Adult	16 (15.2)	32 (30.5)	22 (21.0)	29 (27.6)	6 (5.7)	105		
	16–49 Pregnant/lactating	5 (17.2)	5 (17.2)	5 (17.2)	10 (34.5)	4 (13.8)	29		
	Total	49 (12.4)	122 (30.8)	94 (23.7)	111 (28.0)	20 (5.1)	396		

SD, Strongly Disagree; D, Disagree; N, Neutral; A, Agree; SA, Strongly Agree; χ^2^, Pearson Chi-square statistic. Percentages are presented within each age category. A *p*-value <0.05 indicates a statistically significant association between age category (16–25 undergraduates, ≥25 adults, and 16–49 pregnant/lactating women) and the Suya consumption variable under consideration.

Similarly, night-time consumption (χ^2^ = 12.888, *p* = 0.116), preference for Suya over other meats (χ^2^ = 6.543, *p* = 0.587), and consuming Suya more than other meat snacks (χ^2^ = 5.158, p = 0.741) showed no statistically significant age-related differences. For example, 54.8% of respondents agreed and 24.5% strongly agreed that they eat Suya at night, demonstrating high prevalence across age groups. These findings suggest that such behaviors may be culturally embedded and widely practiced irrespective of age. Comparable studies have reported that street food consumption patterns are commonly driven by shared taste preferences, convenience, and accessibility across demographic groups (30–32).

Buying Suya based on taste rather than hygiene also did not show a significant association with age (χ^2^ = 12.591, *p* = 0.127), although 40.7% of respondents agreed and 7.6% strongly agreed with this practice. Likewise, increased weekend consumption (χ^2^ = 11.015, *p* = 0.201) and consuming Suya because it is readily available (χ^2^ = 10.783, *p* = 0.214) were not age-dependent. These findings indicate that availability, sensory appeal, and routine consumption behaviors are shared across age categories.

In contrast, purchasing Suya from roadside vendors showed a statistically significant association with age category (χ^2^ = 23.307, *p* = 0.003). Overall, 58.3% agreed and 21.0% strongly agreed that they buy from roadside vendors. Agreement was highest among 16–25 undergraduates, suggesting greater reliance on informal food outlets within this group. A similar significant association was observed for patronizing the same vendor (χ^2^ = 23.668, *p* = 0.003), with 40.9% agreeing and 9.6% strongly agreeing overall. Younger respondents demonstrated higher levels of vendor loyalty compared to older adults and pregnant/lactating women.

These significant findings align with evidence that youth and young adults are more likely to patronize street food vendors due to affordability, accessibility, and proximity to schools or residential areas (30–32). However, heavy reliance on roadside vendors raises important public health concerns. Studies from Nigeria and other low- and middle-income countries have consistently documented that inadequate vendor training, limited regulatory oversight, and poor environmental sanitation contribute significantly to food contamination risks (32, 33).

Although most consumption behaviors did not vary significantly by age, their high prevalence remains noteworthy. For instance, nearly 79.3% of respondents agreed and strongly agreed that they consume Suya at night, and 48.3% reported purchasing based on taste rather than hygiene. These patterns may increase exposure to improperly handled meat, particularly in informal vending environments where temperature control and hygiene standards may be inconsistent. Similar patterns have been documented in previous studies where sensory appeal and convenience frequently outweigh food safety considerations (30–32).

Overall, while most Suya consumption behaviors appear broadly consistent across age categories, vendor-related practices differ significantly, particularly among younger respondents. These findings underscore the need for strengthened food safety monitoring and targeted public health interventions within informal street food settings (32, 33).

## Association between occupational category and determinants of Suya consumption

Table 3 presents the association between occupational category and determinants of Suya consumption among 396 respondents in Yenagoa metropolis. The majority of respondents were students (70.7%), followed by civil servants (19.2%), traders (1.3%), artisans (0.5%), unemployed individuals (2.5%), and others (5.8%). Most determinants of Suya consumption did not differ significantly across occupational groups. Perceived affordability showed no statistically significant association with occupation (χ^2^ = 16.667, *p* = 0.674), although 42.4% of respondents agreed and 7.3% strongly agreed that Suya is affordable compared to other meat types. Students (43.6%) and civil servants (38.2%) demonstrated high agreement levels, suggesting affordability is a common driver across occupational groups.

**Table 3:** Association Between Occupational Category and Determinants of Suya Consumption in Yenagoa Metropolis, Bayelsa State, Nigeria.

**Question**	**Occupation**	**SD *n* (%)**	**D *n* (%)**	**N *n* (%)**	**A *n* (%)**	**SA *n* (%)**	**Total**	**χ^2^**	***p*-value**
Suya is affordable compared to other meat types	Student	24 (8.6)	68 (24.3)	44 (15.7)	122 (43.6)	22 (7.9)	280	16.667	0.674
	Trader	1 (20.0)	1 (20.0)	1 (20.0)	2 (40.0)	0 (0.0)	5		
	Civil servant	5 (6.6)	26 (34.2)	12 (15.8)	29 (38.2)	4 (5.3)	76		
	Artisan	0 (0.0)	1 (50.0)	0 (0.0)	1 (50.0)	0 (0.0)	2		
	Unemployed	0 (0.0)	2 (20.0)	1 (10.0)	6 (60.0)	1 (10.0)	10		
	Others	6 (26.1)	4 (17.4)	3 (13.0)	8 (34.8)	2 (8.7)	23		
	Total	36 (9.1)	102 (25.8)	61 (15.4)	168 (42.4)	29 (7.3)	396		
Suya is convenient and does not require cooking at home	Student	11 (3.9)	28 (10.0)	47 (16.8)	162 (57.9)	32 (11.4)	280	16.240	0.702
	Trader	0 (0.0)	0 (0.0)	0 (0.0)	5 (100.0)	0 (0.0)	5		
	Civil servant	2 (2.6)	6 (7.9)	10 (13.2)	44 (57.9)	14 (18.4)	76		
	Artisan	0 (0.0)	0 (0.0)	0 (0.0)	2 (100.0)	0 (0.0)	2		
	Unemployed	0 (0.0)	0 (0.0)	0 (0.0)	8 (80.0)	2 (20.0)	10		
	Others	1 (4.3)	1 (4.3)	1 (4.3)	15 (65.2)	5 (21.7)	23		
	Total	14 (3.5)	35 (8.8)	58 (14.6)	236 (59.6)	53 (13.4)	396		
The aroma and spice of Suya make it appealing	Student	4 (1.4)	11 (3.9)	42 (15.0)	172 (61.4)	51 (18.2)	280	22.837	0.297
	Trader	0 (0.0)	1 (20.0)	1 (20.0)	3 (60.0)	0 (0.0)	5		
	Civil servant	2 (2.6)	6 (7.9)	4 (5.3)	50 (65.8)	14 (18.4)	76		
	Artisan	0 (0.0)	0 (0.0)	0 (0.0)	0 (0.0)	2 (100.0)	2		
	Unemployed	0 (0.0)	1 (10.0)	0 (0.0)	7 (70.0)	2 (20.0)	10		
	Others	1 (4.3)	1 (4.3)	3 (13.0)	15 (65.2)	3 (13.0)	23		
	Total	7 (1.8)	20 (5.1)	50 (12.6)	247 (62.4)	72 (18.2)	396		
Peer influence encourages me to consume Suya	Student	77 (27.5)	109 (38.9)	49 (17.5)	38 (13.6)	7 (2.5)	280	10.699	0.954
	Trader	2 (40.0)	2 (40.0)	0 (0.0)	1 (20.0)	0 (0.0)	5		
	Civil servant	17 (22.4)	37 (48.7)	9 (11.8)	11 (14.5)	2 (2.6)	76		
	Artisan	1 (50.0)	1 (50.0)	0 (0.0)	0 (0.0)	0 (0.0)	2		
	Unemployed	2 (20.0)	4 (40.0)	1 (10.0)	3 (30.0)	0 (0.0)	10		
	Others	8 (34.8)	6 (26.1)	5 (21.7)	3 (13.0)	1 (4.3)	23		
	Total	107 (27.0)	159 (40.2)	64 (16.2)	56 (14.1)	10 (2.5)	396		
I consume Suya because of its availability near my workplace or school	Student	31 (11.1)	84 (30.0)	51 (18.2)	102 (36.4)	12 (4.3)	280	27.960	0.110
	Trader	1 (20.0)	2 (40.0)	0 (0.0)	2 (40.0)	0 (0.0)	5		
	Civil servant	12 (15.8)	30 (39.5)	16 (21.1)	13 (17.1)	5 (6.6)	76		
	Artisan	0 (0.0)	2 (100.0)	0 (0.0)	0 (0.0)	0 (0.0)	2		
	Unemployed	0 (0.0)	4 (40.0)	2 (20.0)	3 (30.0)	1 (10.0)	10		
	Others	6 (26.1)	9 (39.1)	6 (26.1)	2 (8.7)	0 (0.0)	23		
	Total	50 (12.6)	131 (33.1)	75 (18.9)	122 (30.8)	18 (4.5)	396		
I trust the vendors who prepare the Suya I eat	Student	35 (12.5)	91 (32.5)	106 (37.9)	42 (15.0)	6 (2.1)	280	26.118	0.162
	Trader	1 (20.0)	1 (20.0)	1 (20.0)	2 (40.0)	0 (0.0)	5		
	Civil servant	19 (25.0)	32 (42.1)	16 (21.1)	9 (11.8)	0 (0.0)	76		
	Artisan	0 (0.0)	0 (0.0)	2 (100.0)	0 (0.0)	0 (0.0)	2		
	Unemployed	2 (20.0)	4 (40.0)	2 (20.0)	2 (20.0)	0 (0.0)	10		
	Others	5 (21.7)	10 (43.5)	7 (30.4)	1 (4.3)	0 (0.0)	23		
	Total	62 (15.7)	138 (34.8)	134 (33.8)	56 (14.1)	6 (1.5)	396		
I consider Suya as a quick solution when I am hungry	Student	49 (17.5)	100 (35.7)	65 (23.2)	59 (21.1)	7 (2.5)	280	21.742	0.355
	Trader	1 (20.0)	2 (40.0)	0 (0.0)	2 (40.0)	0 (0.0)	5		
	Civil servant	10 (13.2)	35 (46.1)	15 (19.7)	14 (18.4)	2 (2.6)	76		
	Artisan	0 (0.0)	1 (50.0)	0 (0.0)	1 (50.0)	0 (0.0)	2		
	Unemployed	1 (10.0)	4 (40.0)	2 (20.0)	3 (30.0)	0 (0.0)	10		
	Others	7 (30.4)	9 (39.1)	2 (8.7)	2 (8.7)	3 (13.0)	23		
	Total	68 (17.2)	151 (38.1)	84 (21.2)	81 (20.5)	12 (3.0)	396		
I do not think much about the source of the meat used in Suya	Student	41 (14.6)	58 (20.7)	55 (19.6)	110 (39.3)	16 (5.7)	280	15.967	0.719
	Trader	0 (0.0)	1 (20.0)	0 (0.0)	4 (80.0)	0 (0.0)	5		
	Civil servant	9 (11.8)	21 (27.6)	10 (13.2)	28 (36.8)	8 (10.5)	76		
	Artisan	0 (0.0)	1 (50.0)	0 (0.0)	1 (50.0)	0 (0.0)	2		
	Unemployed	1 (10.0)	1 (10.0)	2 (20.0)	4 (40.0)	2 (20.0)	10		
	Others	5 (21.7)	4 (17.4)	3 (13.0)	10 (43.5)	1 (4.3)	23		
	Total	56 (14.1)	86 (21.7)	70 (17.7)	157 (39.6)	27 (6.8)	396		
I rarely inquire about the hygienic practices of Suya vendors	Student	21 (7.5)	45 (16.1)	51 (18.2)	141 (50.4)	22 (7.9)	280	23.896	0.247
	Trader	0 (0.0)	0 (0.0)	1 (20.0)	3 (60.0)	1 (20.0)	5		
	Civil servant	3 (3.9)	12 (15.8)	12 (15.8)	39 (51.3)	10 (13.2)	76		
	Artisan	0 (0.0)	0 (0.0)	0 (0.0)	1 (50.0)	1 (50.0)	2		
	Unemployed	0 (0.0)	1 (10.0)	1 (10.0)	8 (80.0)	0 (0.0)	10		
	Others	2 (8.7)	6 (26.1)	1 (4.3)	8 (34.8)	6 (26.1)	23		
	Total	26 (6.6)	64 (16.2)	66 (16.7)	200 (50.5)	40 (10.1)	396		
Cultural practices or traditions influence my preference for Suya	Student	67 (23.9)	131 (46.8)	56 (20.0)	24 (8.6)	2 (0.7)	280	9.015	0.983
	Trader	2 (40.0)	3 (60.0)	0 (0.0)	0 (0.0)	0 (0.0)	5		
	Civil servant	17 (22.4)	39 (51.3)	14 (18.4)	6 (7.9)	0 (0.0)	76		
	Artisan	1 (50.0)	0 (0.0)	1 (50.0)	0 (0.0)	0 (0.0)	2		
	Unemployed	3 (30.0)	4 (40.0)	1 (10.0)	2 (20.0)	0 (0.0)	10		
	Others	7 (30.4)	9 (39.1)	5 (21.7)	2 (8.7)	0 (0.0)	23		
	Total	97 (24.5)	186 (47.0)	77 (19.4)	34 (8.6)	2 (0.5)	396		

SD, Strongly Disagree; D, Disagree; N, Neutral; A, Agree; SA, Strongly Agree; χ^2^, Pearson Chi-square statistic. Frequencies (*n*) and percentages (%) are presented within each occupational category. Pearson chi-square tests assessed the association between occupational category and each determinant of Suya consumption. A *p*-value <0.05 indicates a statistically significant association between occupation and the variable under consideration.

Similarly, convenience (defined as not requiring home preparation) was widely acknowledged (χ^2^ = 16.240, *p* = 0.702), with 59.6% agreeing and 13.4% strongly agreeing overall. Among students, 57.9% agreed and 11.4% strongly agreed, while 57.9% of civil servants agreed. The aroma and spice of Suya were also strong motivators (χ^2^ = 22.837, *p* = 0.297), with 62.4% agreeing and 18.2% strongly agreeing overall, indicating broad sensory appeal across occupations.

Peer influence did not significantly vary by occupation (χ^2^ = 10.699, *p* = 0.954), with 67.2% either disagreeing and strongly disagreeing that peer pressure encourages consumption. Likewise, perceiving Suya as a quick solution when hungry showed no significant association (χ^2^ = 21.742, *p* = 0.355), although 20.5% agreed and 3.0% strongly agreed overall.

Availability of Suya near the workplace and school also did not demonstrate a statistically significant association in this analysis (χ^2^ = 27.960, *p* = 0.110). Nonetheless, 30.8% agreed and 4.5% strongly agreed that proximity influences their consumption. Agreement was particularly notable among students (36.4%) and civil servants (17.1%), reflecting the clustering of Suya vendors around educational institutions and workplaces.

Food safety–related behaviors similarly showed no significant occupational differences. Notably, 39.6% of respondents agreed and 6.8% strongly agreed that they do not think much about the source of the meat used in Suya (χ^2^ = 15.967, *p* = 0.719). Furthermore, 50.5% agreed and 10.1% strongly agreed that they rarely inquire about vendors' hygienic practices (χ^2^ = 23.896, *p* = 0.247). Trust in vendors also did not significantly differ across occupations (χ^2^ = 26.118, *p* = 0.162), with 33.8% responding neutrally and only 14.1% agreeing. Cultural influence on preference was similarly uniform (χ^2^ = 9.015, *p* = 0.983), with 47.0% disagreeing that tradition drives their preference.

These findings suggest that, although occupational distribution differs within the study population, determinants of Suya consumption are largely consistent across groups. Accessibility, affordability, convenience, and sensory appeal remain dominant motivators irrespective of employment category. This pattern aligns with evidence that street food patronage in urban settings is strongly shaped by convenience and spatial proximity, particularly around schools and workplaces (23, 34). Additionally, the widespread agreement regarding aroma, spice, and convenience supports findings that street foods are valued across social groups for sensory appeal and ease of access (22, 35, 36).

The uniformity in food safety risk perception across occupations is noteworthy. High proportions of respondents reported not considering meat source (39.6%) and rarely inquiring about hygiene practices (60.6% combined agree/strongly agree), indicating limited safety vigilance across employment categories. Such generalized low-risk perception may contribute to previously documented microbial contamination and trace metal presence in Suya (10, 20). Overall, while occupation does not significantly alter most determinants of Suya consumption, shared cultural preferences, convenience, and limited food safety scrutiny appear to drive consumption patterns across all occupational groups.

## Association between educational level and knowledge of trace metal contamination and health concerns related to Suya consumption

The association between educational level (Secondary, Tertiary, Postgraduate) and knowledge of trace metal contamination and health concerns related to Suya consumption is presented in Table 4. For basic awareness, differences across educational groups were not statistically significant. For example, having heard about the possibility of metal contamination in Suya was not associated with education (χ^2^ = 5.697, *p* = 0.681). Across all respondents, 43.2% agreed and strongly agreed, while 37.1% disagreed and strongly disagreed. Similarly, awareness that environmental pollution can lead to metal buildup in meat showed no significant association (χ^2^ = 10.116, *p* = 0.257), yet agreement was high overall, with 63.6% agreeing and strongly agreeing. General knowledge that trace metals (e.g., zinc, chromium, nickel) can be harmful in high amounts also did not differ significantly by education (χ^2^ = 11.572, *p* = 0.171), with a very high 79.1% agreeing and strongly agreeing. These results suggest that general contamination awareness is widely shared, likely spread through informal and mass communication channels (e.g., radio, social media, interpersonal networks), rather than formal education pathways.

**Table 4:** Association between educational level and knowledge of trace metal contamination and health risks related to Suya consumption in Yenagoa Metropolis, Nigeria.

**Question**	**Educational level**	**SD *n* (%)**	**D *n* (%)**	**N *n* (%)**	**A *n* (%)**	**SA *n* (%)**	**Total**	**χ^2^**	***p*-value**
Heard about possibility of metal contamination in Suya	Secondary	2 (15.4)	5 (38.5)	1 (7.7)	4 (30.8)	1 (7.7)	13	5.697	0.681
	Tertiary	23 (8.1)	79 (27.8)	61 (21.5)	105 (37.0)	16 (5.6)	284		
	Postgraduate	9 (9.1)	29 (29.3)	16 (16.2)	35 (35.4)	10 (10.1)	99		
	Total	34 (8.6)	113 (28.5)	78 (19.7)	144 (36.4)	27 (6.8)	396		
Aware environmental pollution can cause metal buildup in meat	Secondary	1 (7.7)	2 (15.4)	2 (15.4)	7 (53.8)	1 (7.7)	13	10.116	0.257
	Tertiary	8 (2.8)	52 (18.3)	54 (19.0)	143 (50.4)	27 (9.5)	284		
	Postgraduate	3 (3.0)	10 (10.1)	12 (12.1)	58 (58.6)	16 (16.2)	99		
	Total	12 (3.0)	64 (16.2)	68 (17.2)	208 (52.5)	44 (11.1)	396		
Know trace metals (zinc, chromium, nickel etc.) can be harmful in high amounts	Secondary	0 (0.0)	0 (0.0)	2 (15.4)	7 (53.8)	4 (30.8)	13	11.572	0.171
	Tertiary	13 (4.6)	17 (6.0)	32 (11.3)	163 (57.4)	59 (20.8)	284		
	Postgraduate	2 (2.0)	11 (11.1)	6 (6.1)	50 (50.5)	30 (30.3)	99		
	Total	15 (3.8)	28 (7.1)	40 (10.1)	220 (55.6)	93 (23.5)	396		
Read/seen information on food contamination in Nigeria	Secondary	0 (0.0)	1 (7.7)	5 (38.5)	4 (30.8)	3 (23.1)	13	37.486	0.000
	Tertiary	5 (1.8)	22 (7.7)	22 (7.7)	182 (64.1)	53 (18.7)	284		
	Postgraduate	1 (1.0)	7 (7.1)	4 (4.0)	47 (47.5)	40 (40.4)	99		
	Total	6 (1.5)	30 (7.6)	31 (7.8)	233 (58.8)	96 (24.2)	396		
Understand Suya may pose long-term health risks if contaminated	Secondary	1 (7.7)	0 (0.0)	2 (15.4)	7 (53.8)	3 (23.1)	13	16.633	0.034
	Tertiary	7 (2.5)	16 (5.6)	45 (15.8)	163 (57.4)	53 (18.7)	284		
	Postgraduate	1 (1.0)	7 (7.1)	8 (8.1)	48 (48.5)	35 (35.4)	99		
	Total	9 (2.3)	23 (5.8)	55 (13.9)	218 (55.1)	91 (23.0)	396		
Know how trace-metal contaminated meat affects health (kidney, liver, cancer)	Secondary	0 (0.0)	1 (7.7)	4 (30.8)	6 (46.2)	2 (15.4)	13	13.056	0.110
	Tertiary	10 (3.5)	23 (8.1)	49 (17.3)	160 (56.3)	42 (14.8)	284		
	Postgraduate	3 (3.0)	9 (9.1)	14 (14.1)	44 (44.4)	29 (29.3)	99		
	Total	13 (3.3)	33 (8.3)	67 (16.9)	210 (53.0)	73 (18.4)	396		
Believe roadside preparation increases contamination risk	Secondary	0 (0.0)	0 (0.0)	2 (15.4)	8 (61.5)	3 (23.1)	13	4.433	0.816
	Tertiary	5 (1.8)	20 (7.0)	57 (20.1)	161 (56.7)	41 (14.4)	284		
	Postgraduate	1 (1.0)	5 (5.1)	16 (16.2)	57 (57.6)	20 (20.2)	99		
	Total	6 (1.5)	25 (6.3)	75 (18.9)	226 (57.1)	64 (16.2)	396		
Understand bioaccumulation of metals in animal tissues	Secondary	0 (0.0)	4 (30.8)	4 (30.8)	3 (23.1)	2 (15.4)	13	23.959	0.002
	Tertiary	13 (4.6)	42 (14.8)	87 (30.6)	122 (43.0)	20 (7.0)	284		
	Postgraduate	2 (2.0)	13 (13.1)	24 (24.2)	37 (37.4)	23 (23.2)	99		
	Total	15 (3.8)	59 (14.9)	115 (29.0)	162 (40.9)	45 (11.4)	396		
Aware of difference between essential vs. toxic levels of trace metals	Secondary	0 (0.0)	3 (23.1)	6 (46.2)	3 (23.1)	1 (7.7)	13	16.222	0.039
	Tertiary	10 (3.5)	45 (15.8)	84 (29.6)	121 (42.6)	24 (8.5)	284		
	Postgraduate	4 (4.0)	13 (13.1)	19 (19.2)	43 (43.4)	20 (20.2)	99		
	Total	14 (3.5)	61 (15.4)	109 (27.5)	167 (42.2)	45 (11.4)	396		
Believe regular health checks of Suya vendors should be enforced	Secondary	1 (7.7)	0 (0.0)	1 (7.7)	6 (46.2)	5 (38.5)	13	18.468	0.018
	Tertiary	2 (0.7)	5 (1.8)	32 (11.3)	148 (52.1)	97 (34.2)	284		
	Postgraduate	4 (4.0)	2 (2.0)	3 (3.0)	42 (42.4)	48 (48.5)	99		
	Total	7 (1.8)	7 (1.8)	36 (9.1)	196 (49.5)	150 (37.9)	396		

SD, Strongly Disagree; D, Disagree; N, Neutral; A, Agree; SA, Strongly Agree. Values are presented as frequency (*n*) and percentage (%) within educational level. χ^2^ = Pearson Chi-Square test of association; *p*-values are two-tailed. Statistical significance was set at *p* < 0.05.

In contrast, education showed strong effects on information exposure and more advanced/analytical understanding. A strong and statistically significant relationship was observed between education and having read/seen information on food contamination in Nigeria (χ^2^ = 37.486, *p* < 0 0.001). Overall agreement was very high (83.0%), but the pattern clearly reflected higher endorsement among more educated respondents, especially postgraduates (87.9%) compared with tertiary (82.8%) and secondary (53.9%). This indicates that higher educational attainment increases the likelihood of being exposed to and engaging with formal food safety information (30, 37–39).

Education was also significantly associated with understanding that contaminated Suya may pose long-term health risks (χ^2^ = 16.633, *p* = 0.034). In the total sample, 78.1% agreed and strongly agreed. Notably, endorsement was highest among postgraduates (83.9%), followed by tertiary (76.1%) and secondary (76.9%). This supports the view that education strengthens the ability to connect contamination risks with chronic outcomes such as kidney and liver damage and cancer, reinforcing evidence that education improves risk perception and health decision-making for environmental and foodborne hazards.

More importantly, education significantly influenced comprehension of technical food safety concepts. Understanding bioaccumulation of metals in animal tissues was significantly associated with educational level (χ^2^ = 23.959, *p* = 0.002). While overall agreement was moderate (52.3%), it varied markedly by education: 60.6% of postgraduates agreed/strongly agreed compared with 50.0% of tertiary respondents and only 38.5% of secondary respondents. Similarly, awareness of the difference between essential and toxic levels of trace metals also differed significantly by education (χ^2^ = 16.222, *p* = 0.039). Although overall agreement was again moderate (53.6%), it was higher among postgraduates (63.6%) than among tertiary (51.1%) and secondary (30.8%) respondents. These results indicate that scientific/technical understanding is unevenly distributed, with higher education conferring an advantage in interpreting complex contamination concepts (40, 41).

Support for regulatory action also showed significant educational patterning. Belief that regular health checks of Suya vendors should be enforced was significantly associated with education (χ^2^ = 18.468, *p* =0.018), and agreement was exceptionally high across the sample (87.4%). However, support was strongest among postgraduates (90.9%) and secondary respondents (84.7%), with tertiary respondents similarly high (86.3%). This suggests that while support for vendor regulation is broadly shared, higher education tends to align with stronger endorsement of institutional and regulatory interventions for street food safety.

Finally, education did not significantly influence some perception items that may be shaped by common experience rather than schooling. For example, belief that roadside preparation increases contamination risk was not significant (χ^2^ = 4.433, *p* = 0.816), yet overall agreement was high (73.3%). Likewise, knowledge of how contaminated meat affects health (kidney, liver, cancer) was not significantly associated with education (χ^2^ = 13.056, *p* = 0.110), but agreement still exceeded 71.4%. This suggests that experiential knowledge and shared public narratives about street foods may override educational differences for broad risk beliefs.

Overall, education is a significant determinant of advanced, technical, and regulatory-oriented food safety knowledge, while basic awareness and general risk perceptions are broadly shared across educational levels. This pattern stresses the importance of targeted risk communication strategies that translate complex scientific concepts (e.g., bioaccumulation and toxic thresholds) into clear, accessible messages for consumers at all educational levels. While also strengthening public engagement with credible food safety information sources.

## Association between educational level and awareness, risk perception, and safety practices related to Suya consumption

Table 5 presents the association between educational attainment (Secondary, Tertiary, and Postgraduate) and awareness, risk perception, and safety practices related to Suya consumption among 396 respondents in Yenagoa metropolis. Concern about the hygiene of Suya preparation did not differ significantly across educational levels (χ^2^ = 8.816, *p* = 0.358). Overall, 55.8% agreed and 16.7% strongly agreed that they were concerned about hygiene, indicating that 72.5% of respondents expressed concern irrespective of education. Similarly, willingness to reduce Suya intake if it contained toxic metals showed no significant association (χ^2^ = 10.284, *p* = 0.246), although 48.0% agreed and 32.8% strongly agreed, suggesting that over 80% would modify consumption under perceived risk.

**Table 5:** Association between educational level and awareness, risk perception, and safety practices related to Suya consumption in Yenagoa Metropolis, Nigeria.

**Question**	**Educational level**	**SD *n* (%)**	**D *n* (%)**	**N *n* (%)**	**A *n* (%)**	**SA *n* (%)**	**Total**	**χ^2^**	***p*-value**
Concerned about the hygiene of Suya preparation in my area	Secondary	0 (0.0)	1 (7.7)	3 (23.1)	5 (38.5)	4 (30.8)	13	8.816	0.358
	Tertiary	2 (0.7)	15 (5.3)	62 (21.8)	163 (57.4)	42 (14.8)	284		
	Postgraduate	3 (3.0)	7 (7.1)	16 (16.2)	53 (53.5)	20 (20.2)	99		
	Total	5 (1.3)	23 (5.8)	81 (20.5)	221 (55.8)	66 (16.7)	396		
Would reduce Suya intake if I knew it contained toxic metals	Secondary	0 (0.0)	1 (7.7)	2 (15.4)	3 (23.1)	7 (53.8)	13	10.284	0.246
	Tertiary	7 (2.5)	9 (3.2)	43 (15.1)	142 (50.0)	83 (29.2)	284		
	Postgraduate	2 (2.0)	1 (1.0)	11 (11.1)	45 (45.5)	40 (40.4)	99		
	Total	9 (2.3)	11 (2.8)	56 (14.1)	190 (48.0)	130 (32.8)	396		
Believe health authorities should monitor the safety of Suya sold in the metropolis	Secondary	0 (0.0)	0 (0.0)	0 (0.0)	7 (53.8)	6 (46.2)	13	26.485	0.001
	Tertiary	1 (0.4)	4 (1.4)	27 (9.5)	154 (54.2)	98 (34.5)	284		
	Postgraduate	4 (4.0)	0 (0.0)	4 (4.0)	36 (36.4)	55 (55.6)	99		
	Total	5 (1.3)	4 (1.0)	31 (7.8)	197 (49.7)	159 (40.2)	396		
Have never received education/public information about contaminated Suya	Secondary	1 (7.7)	2 (15.4)	1 (7.7)	5 (38.5)	4 (30.8)	13	12.537	0.129
	Tertiary	10 (3.5)	56 (19.7)	51 (18.0)	122 (43.0)	45 (15.8)	284		
	Postgraduate	7 (7.1)	16 (16.2)	10 (10.1)	39 (39.4)	27 (27.3)	99		
	Total	18 (4.5)	74 (18.7)	62 (15.7)	166 (41.9)	76 (19.2)	396		
Support testing Suya samples for safety in my community	Secondary	0 (0.0)	0 (0.0)	1 (7.7)	6 (46.2)	6 (46.2)	13	10.688	0.220
	Tertiary	4 (1.4)	5 (1.8)	42 (14.8)	145 (51.1)	88 (31.0)	284		
	Postgraduate	1 (1.0)	1 (1.0)	6 (6.1)	46 (46.5)	45 (45.5)	99		
	Total	5 (1.3)	6 (1.5)	49 (12.4)	197 (49.7)	139 (35.1)	396		
Wash Suya with water or reheat it before eating (if taken home)	Secondary	6 (46.2)	5 (38.5)	1 (7.7)	1 (7.7)	0 (0.0)	13	9.420	0.308
	Tertiary	67 (23.6)	143 (50.4)	47 (16.5)	20 (7.0)	7 (2.5)	284		
	Postgraduate	29 (29.3)	46 (46.5)	9 (9.1)	11 (11.1)	4 (4.0)	99		
	Total	102 (25.8)	194 (49.0)	57 (14.4)	32 (8.1)	11 (2.8)	396		
Prefer to buy Suya from certified vendors if available	Secondary	0 (0.0)	1 (7.7)	1 (7.7)	6 (46.2)	5 (38.5)	13	11.398	0.180
	Tertiary	5 (1.8)	12 (4.2)	41 (14.4)	153 (53.9)	73 (25.7)	284		
	Postgraduate	4 (4.0)	3 (3.0)	8 (8.1)	45 (45.5)	39 (39.4)	99		
	Total	9 (2.3)	16 (4.0)	50 (12.6)	204 (51.5)	117 (29.5)	396		
Willing to stop eating Suya if proven to pose a health risk	Secondary	1 (7.7)	1 (7.7)	1 (7.7)	4 (30.8)	6 (46.2)	13	29.407	0.000
	Tertiary	7 (2.5)	20 (7.0)	61 (21.5)	130 (45.8)	66 (23.2)	284		
	Postgraduate	2 (2.0)	6 (6.1)	9 (9.1)	33 (33.3)	49 (49.5)	99		
	Total	10 (2.5)	27 (6.8)	71 (17.9)	167 (42.2)	121 (30.6)	396		
Think more public awareness campaigns are needed on food safety	Secondary	0 (0.0)	0 (0.0)	1 (7.7)	6 (46.2)	6 (46.2)	13	13.428	0.098
	Tertiary	2 (0.7)	3 (1.1)	16 (5.6)	122 (43.0)	141 (49.6)	284		
	Postgraduate	1 (1.0)	0 (0.0)	0 (0.0)	32 (32.3)	66 (66.7)	99		
	Total	3 (0.8)	3 (0.8)	17 (4.3)	160 (40.4)	213 (53.8)	396		
Want to know more about how to protect myself from contaminated food	Secondary	0 (0.0)	0 (0.0)	0 (0.0)	6 (46.2)	7 (53.8)	13	18.668	0.017
	Tertiary	1 (0.4)	2 (0.7)	22 (7.7)	149 (52.5)	110 (38.7)	284		
	Postgraduate	2 (2.0)	0 (0.0)	2 (2.0)	37 (37.4)	58 (58.6)	99		
	Total	3 (0.8)	2 (0.5)	24 (6.1)	192 (48.5)	175 (44.2)	396		

SD, Strongly Disagree; D, Disagree; N, Neutral; A, Agree; SA, Strongly Agree; χ^2^, Pearson Chi-square statistic. Frequencies (*n*) and percentages (%) are presented within each educational level. Pearson chi-square tests assessed the association between educational attainment (Secondary, Tertiary, and Postgraduate) and each awareness, risk perception, and safety practice variable related to Suya consumption. A *p*-value <0.05 indicates a statistically significant association between educational level and the variable under consideration.

In contrast, a statistically significant association was observed between educational level and the belief that health authorities should monitor Suya safety (χ^2^ = 26.485, *p* = 0.001). Overall, 49.7% agreed and 40.2% strongly agreed, indicating strong consensus (89.9%). Agreement was particularly high among postgraduate respondents, 55.6% of whom strongly agreed. This suggests that higher education enhances recognition of institutional responsibility in food safety governance. Similar findings have been reported in studies linking educational exposure with increased confidence in food safety regulations and oversight mechanisms (39, 42, 43).

Educational attainment also significantly influenced willingness to stop eating Suya if proven to pose a health risk (χ^2^ = 29.407, *p* < 0.001). Overall, 42.2% agreed and 30.6% strongly agreed, meaning 72.8% expressed readiness to discontinue consumption. Strong agreement was highest among postgraduate respondents (49.5%). This indicates that education may strengthen risk-responsive behavior, supporting evidence that higher educational attainment improves risk interpretation and promotes adaptive dietary choices (44).

Similarly, desire to learn more about protecting oneself from contaminated food was significantly associated with education (χ^2^ = 18.668, *p* = 0.017). A combined 92.7% of respondents agreed or strongly agreed (48.5% agree; 44.2% strongly agree), with postgraduate respondents again showing high levels of strong agreement (58.6%). This reflects education's role in fostering proactive, health-seeking attitudes.

Other variables showed no significant educational differences despite high levels of overall agreement. Support for testing Suya samples (χ^2^ = 10.688, *p* = 0.220) was substantial, with 49.7% agreeing and 35.1% strongly agreeing (84.8% combined). Preference for certified vendors (χ^2^ = 11.398, *p* = 0.180) was also widely endorsed (51.5% agree; 29.5% strongly agree). These findings suggest that support for regulatory measures is broadly shared across educational groups.

Routine safety practices, such as washing and reheating Suya before consumption, did not significantly differ by education (χ^2^ = 9.420, *p* = 0.308). Notably, 25.8% strongly disagreed and 49.0% disagreed with engaging in this practice, indicating that 74.8% do not routinely reheat and wash Suya before eating. This uniformity suggests that habitual practices may be resistant to change, even among more educated respondents.

Similarly, having never received public information about contaminated Suya was not significantly associated with education (χ^2^ = 12.537, *p* = 0.129), although 41.9% agreed and 19.2% strongly agreed. The perception that more public awareness campaigns are needed also showed no statistically significant difference (χ^2^ = 13.428, *p* = 0.098), despite strong overall support (40.4% agree; 53.8% strongly agree).

Overall, the findings indicate that educational attainment plays a selective but important role in shaping higher-order awareness and regulatory-oriented perceptions, particularly regarding institutional monitoring and risk-responsive behavior. However, basic hygiene concerns and routine safety practices appear to be widely shared across educational categories. Education therefore influences advanced risk interpretation and institutional trust more strongly than everyday consumption habits.

## Internal consistency of the Suya consumption and food safety questionnaire

The internal consistency of the Suya consumption and food safety questionnaire was assessed using Cronbach's alpha (Table 6). The results show that all sections demonstrated acceptable to high reliability, with values ranging from 0.709 to 0.856 and an overall alpha of 0.825. Section C, which assessed knowledge of trace-metal contamination, had the highest reliability (α = 0.856). This indicates that the items were highly consistent in measuring respondents' knowledge about the health risks associated with contaminated Suya. This suggests that participants understood the questions clearly and responded consistently, reflecting the robustness of the knowledge assessment component.

**Table 6:** Internal consistency of the Suya consumption and food safety questionnaire sections assessed by Cronbach's alpha.

**Section**	**Description**	**No. of items**	***N***	**Cronbach's α**
A	Suya consumption patterns	9	396	0.815
B	Factors influencing consumption	10	396	0.709
C	Knowledge of trace metal contamination	10	396	0.856
D	Awareness and safety practices	10	396	0.788
–	Overall questionnaire	39	396	0.825

Cronbach's alpha values indicate acceptable to good internal consistency for all sections and the overall questionnaire.

Section A (Suya Consumption Patterns) also demonstrated high reliability (α = 0.815), confirming that respondents' reported consumption behaviors were measured consistently. Section D (Awareness and Safety Practices) showed good reliability (α = 0.788), indicating that responses on safety practices and perceptions were coherent and comparable across participants. Section B (Factors Influencing Consumption) had the lowest reliability (α = 0.709) among the four sections, although still within the acceptable range (27). The slightly lower alpha may reflect variability in individual perceptions of influencing factors, such as peer pressure, affordability, or convenience, which are more subjective and can differ widely among respondents.

The Cronbach's alpha results confirm that the questionnaire is a reliable tool for measuring Suya consumption, influencing factors, knowledge, and safety practices, and that the observed variations, particularly in knowledge and consumption patterns, provide meaningful insights into behaviors and perceptions related to trace metal contamination.

## Association among Suya consumption patterns, factors influencing consumption, knowledge of trace metal contamination, and awareness/safety practices using Spearman's rank correlation

Table 7 presents the associations among Suya consumption patterns, factors influencing consumption, knowledge of trace metal contamination, and awareness/safety practices, using Spearman's rank correlation. The results showed a strong positive correlation between Suya consumption patterns (Section A) and factors influencing consumption (Section B) (*r* = 0.546, *p* < 0.01). This indicates that respondents' consumption behaviors are closely associated with motivating factors such as convenience, affordability, taste, and social influences. This suggests that interventions aimed at modifying consumption patterns should consider the underlying social and environmental drivers that encourage Suya intake.

**Table 7:** Spearman's rank correlations among Suya consumption, influencing factors, knowledge, and safety practices.

**Sections**	**Sections A**	**Sections B**	**Sections C**	**Sections D**
Sections A	1.000			
Sections B	0.546^**^	1.000		
Sections C	−0.050	−0.005	1.000	
Sections D	−0.028	0.026	0.132^**^	1.000

Sections A, Suya Consumption Patterns; Sections B, Factors Influencing Consumption; Sections C, Knowledge of Trace Metal Contamination; Sections D, Awareness/Safety Practices. ^**^Correlation is significant at the 0.01 level (2-tailed).

Knowledge of trace metal contamination (Section C) was weakly and negatively correlated with consumption patterns (*r* = −0.050) and influencing factors (*r* = −0.005), and the relationships were not statistically significant. This implies that despite some awareness of potential contamination, knowledge alone may not strongly influence consumption behaviors, stressing a gap between knowledge and practice.

Awareness and safety practices (Section D) showed a significant positive correlation with knowledge (*r* = 0.132, *p* < 0.01). This suggests that greater awareness of trace metal risks is associated with slightly improved safety behaviors, such as washing, reheating, or selecting safer vendors. The lack of significant correlation between awareness and consumption patterns or influencing factors indicates that even knowledgeable consumers may still engage in high-frequency consumption. This stresses the need for targeted interventions that combine education with behavioral change strategies.

## Conclusion

This study focused on assessing Suya consumption patterns, factors influencing consumption, knowledge of trace metal contamination, and awareness/safety practices among residents of Yenagoa metropolis, Nigeria. The findings revealed that Suya consumption at least once per week is relatively low (approximately 16%), with most respondents being young, female, single, and tertiary students. Significant associations were observed between age and certain consumption behaviors, as well as between occupation and factors influencing Suya consumption, indicating that accessibility and convenience largely drive patronage. Knowledge of trace metal contamination was generally moderate, with higher awareness associated with educational attainment, while both knowledge and perceptions influenced safety practices. Internal consistency of the questionnaire was acceptable to high (Cronbach's α = 0.709–0.856), and correlation analysis showed moderate associations between consumption patterns and influencing factors.

In contrast, knowledge and safety practices were positively but weakly related. These results highlight gaps between awareness and actual consumption behaviors, emphasizing the need for targeted interventions. Based on the findings, there is a need for public education campaigns (through workshops, media programs, and school-based education) to raise awareness of the health risks associated with contaminated Suya and promote safe consumption practices. Food regulatory bodies should monitor and enforce hygienic preparation and compliance with food safety standards.

## Data Availability

The raw data supporting the conclusions of this article will be made available by the authors, without undue reservation.
