# Supplementary material for: Patterns of Suya (roasted beef) consumption, awareness of trace metal contamination, and food safety practices among residents of Yenagoa Metropolis, Nigeria
Source: Front Public Health. 2026 Mar 20;14:1779695. doi: 10.3389/fpubh.2026.1779695 (PMC13047124; doi:10.3389/fpubh.2026.1779695)
Supplement: Supplementary file 1 [file Table_1.docx]

**Patterns of Suya (Roasted Beef) Consumption, Awareness of Trace Metal Contamination, and Food Safety Practices among Residents of Yenagoa Metropolis, Nigeria**

**Sylvester Chibueze Izah**

Department of Community Medicine, Faculty of Clinical Sciences, Bayelsa Medical University, Yenagoa, Bayelsa, Nigeria.

Email: [chivestizah@gmail.com](mailto:chivestizah@gmail.com)

**PART 1: Socioeconomic Characteristics and Awareness of Suya Contaminants**

**Supplementary Material 1: Socio-Demographic Characteristics of Respondents in Yenagoa Metropolis, Bayelsa State, Nigeria**

| Questions | Categories |
| --- | --- |
| Gender | Male |
|  | Female |
|  | Total |
| Age | 16–25 Undergraduate |
|  | ≥ 25 adults |
|  | 16–49 Pregnant/lactating women |
|  | Total |
| Marital Status | Single |
|  | Married |
|  | Widowed |
|  | Total |
| Educational Level | No primary education |
|  | Primary |
|  | Secondary |
|  | Tertiary |
|  | Postgraduate |
|  | Total |
| Occupation | Student |
|  | Trader |
|  | Civil Servant |
|  | Artisan |
|  | Unemployed |
|  | Others |
|  | Total |
| Religion | Christianity |
|  | Islam |
|  | Traditional |
|  | Total |

### PART 2: Consumption Patterns and Associated **Health Risk of Trace Metals in Suya in Yenagoa metropolis, Nigeria**

**Supplementary Material 2: Distribution of Suya Consumption Patterns among Residents of Yenagoa Metropolis, Nigeria**

| **Questions** | **1 = Strongly Disagree** | **2 = Disagree** | **3 = Neutral** | **4 = Agree** | **5 = Strongly Agree** |
| --- | --- | --- | --- | --- | --- |
| I consume suya at least once a week. |  |  |  |  |  |
| I usually buy suya from roadside vendors or local spots. |  |  |  |  |  |
| I often eat suya at afternoon and evening |  |  |  |  |  |
| I prefer suya over other types of cooked meat. |  |  |  |  |  |
| I consume suya more than any other meat snack. |  |  |  |  |  |
| I buy suya based on taste rather than hygiene or safety. |  |  |  |  |  |
| I consume suya more frequently during weekends. |  |  |  |  |  |
| I often patronize the same Suya vendor. |  |  |  |  |  |
| I consume suya because it is readily available in my area. |  |  |  |  |  |

**Supplementary 3: Factors Influencing Suya Consumption among Residents of Yenagoa Metropolis, Nigeria**

| **Questions** | **1 = Strongly Disagree** | **2 = Disagree** | **3 = Neutral** | **4 = Agree** | **5 = Strongly Agree** |
| --- | --- | --- | --- | --- | --- |
| Suya is affordable compared to other meat types. |  |  |  |  |  |
| Suya is convenient and does not require cooking at home. |  |  |  |  |  |
| The aroma and spice of suya make it appealing. |  |  |  |  |  |
| Peer influence encourages me to consume suya. |  |  |  |  |  |
| I consume suya because of its availability near my workplace or school. |  |  |  |  |  |
| I trust the vendors who prepare the suya I eat. |  |  |  |  |  |
| I consider suya as a quick solution when I am hungry. |  |  |  |  |  |
| I do not think much about the source of the meat used in suya. |  |  |  |  |  |
| I rarely inquire about the hygienic practices of Suya vendors. |  |  |  |  |  |
| Cultural practices or traditions influence my preference for suya. |  |  |  |  |  |

**Table 4: Knowledge of Trace Metal Contamination and Associated Health Concerns among Suya Consumers in Yenagoa Metropolis, Nigeria**

| **Questions** | **1 = Strongly Disagree** | **2 = Disagree** | **3 = Neutral** | **4 = Agree** | **5 = Strongly Agree** |
| --- | --- | --- | --- | --- | --- |
| I have heard about the possibility of metal contamination in suya. |  |  |  |  |  |
| I am aware that environmental pollution can lead to metal buildup in meat. |  |  |  |  |  |
| I know that trace metals like zinc, chromium, nickel, etc., can be harmful in high amounts. |  |  |  |  |  |
| I have read or seen information regarding food contamination in Nigeria. |  |  |  |  |  |
| I understand that suya may pose long-term health risks if contaminated. |  |  |  |  |  |
| I know how meat contaminated by trace metals can affect human health (e.g., the kidneys, liver, and cancer). |  |  |  |  |  |
| I believe that roadside preparation of suya increases the risk of contamination. |  |  |  |  |  |
| I understand the concept of bioaccumulation of metals in animal tissues. |  |  |  |  |  |
| I am aware of the difference between essential and toxic levels of trace metals. |  |  |  |  |  |
| Regular health checks of Suya vendors should be enforced. |  |  |  |  |  |

**Table 5: Awareness, Perception, and Safety Practices Related to Suya Consumption in Yenagoa Metropolis, Nigeria**

| **Questions** | **1 = Strongly Disagree** | **2 = Disagree** | **3 = Neutral** | **4 = Agree** | **5 = Strongly Agree** |
| --- | --- | --- | --- | --- | --- |
| I am concerned about the hygiene of Suya preparation in my area. |  |  |  |  |  |
| I would reduce my Suya intake if I knew it contained toxic metals. |  |  |  |  |  |
| Health authorities should monitor the safety of suya sold in the metropolis. |  |  |  |  |  |
| I have never received education or public information about contaminated suya. |  |  |  |  |  |
| I support testing Suya samples for safety in my community. |  |  |  |  |  |
| I wash suya with water or reheat it before eating (if taken home). |  |  |  |  |  |
| I would prefer to buy suya from certified vendors if available. |  |  |  |  |  |
| I am willing to stop eating suya if proven to pose a health risk. |  |  |  |  |  |
| I think more public awareness campaigns are needed on food safety. |  |  |  |  |  |
| I want to know more about how to protect myself from contaminated food. |  |  |  |  |  |
